# Clinical Outcomes with the Use of Dipeptidyl Peptidase-4 (DPP-4) Inhibitor Among Patients with Diabetes Mellitus and COVID-19: A Systematic Review of Observational Studies

**DOI:** 10.3390/jcm15062117

**Published:** 2026-03-10

**Authors:** Shaden A. Alkhalifah, Walaa A. Alshahrani, Abdulmajeed M. Alshehri, Majed S. Al Yami

**Affiliations:** 1 College of Medicine, King Saud bin Abdulaziz University for Health Sciences (KSAU-HS), Riyadh 14611, Saudi Arabia; shadenalkhalifahi@gmail.com; 2 King Abdullah International Medical Research Center (KAIMRC), Riyadh 11481, Saudi Arabia; alshahraniiwa@gmail.com (W.A.A.);; 3 Department of Pharmacy Practice, College of Pharmacy, King Saud bin Abdulaziz University for Health Sciences (KSAU-HS), Riyadh 11481, Saudi Arabia; 4 Ministry of the National Guard-Health Affairs, Riyadh 14611, Saudi Arabia

**Keywords:** DPP-4i (dipeptidyl peptidase-4 inhibitors), COVID-19, glucose lowering agents, mortality, death

## Abstract

**Background**: Diabetics with coronavirus disease 2019 (COVID-19) manifest more adverse clinical outcomes with elevated rates of death. It has been suggested that the SARS-CoV-2 (severe acute respiratory syndrome coronavirus 2) pathway of entrance into the host cell might be assisted by dipeptidyl peptidase-4 (DPP4), leading to inflammation and cytokine storm, with replication into the airways and unfavorable effects in the lungs. Consequently, the goal of this systematic review is to investigate the most recent data on the effect of DPP-4i (dipeptidyl peptidase-4 inhibitor) medications on clinical outcomes, mainly mortality among COVID-19 patients. **Methods**: By conducting a systematic search using PubMed and the Cochrane library, observational studies were identified to examine the association between DPP-4i medications and clinical outcomes including mortality, intensive care unit and hospital admissions. The methodologies of included studies were assessed utilizing the Newcastle–Ottawa Scale (NOS). **Results**: A total of nineteen studies were included with sample sizes varying from over 100 patients to 2.8 million and variant follow-up durations from 30 days up to discharge or death. Most of the population across the studies had COVID-19 for the first time, and the majority were hospitalized. Similarly, mortality definition varied among studies with different time points consisting of 30-day mortality, in-hospital mortality, or all-cause mortality. The majority of the studies identified no effect on mortality by DPP-4i, while a considerable proportion revealed beneficial effects; only four studies showed increased mortality. **Conclusions**: Real-world data from this review suggested a safe use of DPP-4i among COVID-19 patients; however, randomized clinical trials are required to confirm the beneficial outcomes and safe use.

## 1. Introduction

Coronavirus disease 2019 (COVID-19) is caused by the coronavirus known as severe acute respiratory syndrome coronavirus 2 (SARS-CoV-2). The first case of COVID-19 was discovered in December 2019 and has since expanded worldwide [1]. Globally, there were 44,351,506 confirmed COVID-19 cases in 2020, and over one million deaths [2]. Patients with diabetes show pronounced clinical symptoms and more complications [3]. When compared to people without diabetes, persons with COVID-19 who have diabetes had a twofold higher risk of both death and COVID-19 severity [4].

There are several pathophysiological mechanisms in which COVID-19 disease affects patients, leading to adverse complications. One is through a dysregulated immunological response and increased cytokine production such as interleukin-1 (IL-1), interleukin-6 (IL-6), and tumor necrosis factor-α (TNF-α) [5,6]. Another proposed mechanism related to diabetes is through infecting human pancreatic β-cells, which predisposes patients to impaired insulin production, causing worsened systematic metabolic outcomes and poor glycemic control throughout the disease course [7]. An additional process explaining negative outcomes in diabetes is a high glucose level contributing to an enhanced facilitation of SARS-CoV-2 proliferation in monocytes, which are one of the major innate immunity cells that are most found in the lungs of COVID-19 patients [8]. Diabetes is also linked to weakened innate and adaptive immune responses resulting in persistent hyperglycemic-induced inflammation that plays a major role in end-organ damage [9]. The expression of angiotensin-converting enzyme (ACE) in the lungs and other tissues is elevated, and it is one of the receptors that facilitate viral entry and replication, contributing to the COVID-19 infective mechanism [10]. Comorbid conditions such as cardiovascular disease, hypertension, dyslipidemia, obesity, and insulin resistance are also key contributors that have a critical role in COVID-19 diabetic patients’ prognosis [11]. Dipeptidyl peptidase-4 (DPP4) has been proposed as a potential facilitator of SARS-CoV-2 entry into host cells. However, its involvement in viral entry remains uncertain and is less well established than the ACE2-mediated pathway. Another suggested mechanism is that DPP-4 in the lungs may contribute to inflammation and cytokine storm with replication of the virus into the airways; as a result, preventing this interaction has been suggested to improve COVID-19 clinical outcomes [10]. All of these proposed mechanisms regarding DPP-4 need further investigation to establish high-quality evidence. Dipeptidyl peptidase-4 inhibitors (DPP-4i) are one of the antidiabetic medications that could be used for the treatment of COVID-19 disease. DPP-4i medications such as sitagliptin, saxagliptin, linagliptin, vildagliptin, and alogliptin are widely used for their protective glycemic effect by elevating incretin hormones and enhancing insulin secretion [12]. In the context of COVID-19, the proposed non-glycemic effect—associated with their anti-inflammatory action—may involve inhibition of the host CD26 receptor, thereby restricting SARS-CoV-2 entry [10].

Nevertheless, it is still debatable whether COVID-19-infected individuals should continue or cease the intake of some antidiabetic medications. Many studies consisting of observational and case-control papers have assessed the relation between DPP-4i use during COVID-19 infection and medical outcomes. With respect to mortality, the studies demonstrated inconsistent results, varying between protective and neutral effects. Given the heterogeneity and variability of the findings, this gap can be addressed by conducting this updated systematic review, which aims to examine DPP-4i among the COVID-19-infected population and highlight specific findings such as mortality, hospital length of stay, intensive care unit (ICU) admission, and mechanical ventilation.

## 2. Materials and Methods

### 2.1. Search Strategy, Study Selection, and Data Extraction

A systematic search was conducted in PubMed and the Cochrane Library to identify studies evaluating the impact of dipeptidyl peptidase-4 (DPP-4) inhibitor use on clinical outcomes among patients with diabetes mellitus and COVID-19. The PubMed search strategy combined the following terms: “dipeptidyl-peptidase IV inhibitors,” “DPP-4 inhibitors,” “gliptins,” “sitagliptin,” “vildagliptin,” “saxagliptin,” “alogliptin,” or “linagliptin,” paired with “COVID-19,” “SARS-CoV-2 infection,” or “coronavirus disease 2019.” An equivalent strategy was applied to the Cochrane Library from inception to 17 September 2025. Eligible studies included adult patients with diabetes and confirmed COVID-19, compared outcomes between DPP-4 inhibitor users and non-users, and reported at least one relevant endpoint, including all-cause mortality, 30-day mortality, in-hospital mortality, intensive care unit (ICU) admission, mechanical ventilation requirement, hospital length of stay, or ICU length of stay. Studies were excluded if they did not include patients with diabetes and confirmed COVID-19; if they did not evaluate DPP-4 inhibitor exposure; or if they lacked an appropriate comparator group. We also excluded reviews, systematic reviews, meta-analyses, case reports, case series without a control group, conference abstracts, letters without original data, editorials, commentaries, and animal or laboratory-based studies.

### 2.2. Quality Assessment and Risk of Bias

The Newcastle–Ottawa Scale (NOS) evaluates three domains: Selection (0–4 stars), Comparability (0–2 stars), and Outcome (0–3 stars). A maximum score of 9 was used to assess the quality of all included observational studies. Studies that were classified as high quality received 7–9 stars, those scoring 5–6 stars were classified as fair quality, and those with ≤4 stars were classified as low quality [13]. Two reviewers independently conducted the quality assessments, resolving any discrepancies through discussion. This systematic review was reported in accordance with the Preferred Reporting Items for Systematic Reviews and Meta-Analyses (PRISMA) guidelines [14], and the protocol was prospectively registered in the International Prospective Register of Systematic Reviews (PROSPERO; registration number: CRD420251184763). The PRISMA checklist is in the Supplementary Material.

### 2.3. Data Synthesis and Analysis

Due to substantial heterogeneity across the included studies, a meta-analysis could not be performed. The studies differed widely in design, populations, definitions of mortality, and follow-up duration. Therefore, effect estimates (HRs, RRs, ORs, and adjusted measures) were extracted and reported exactly as presented in the original publications without statistical pooling. No subgroup or sensitivity analyses were conducted because of the limited number of studies and their heterogeneity. A structured narrative synthesis was conducted in accordance with PRISMA 2020 guidelines.

## 3. Results

### 3.1. Search Results and Study Characteristics

**Figure 1:**
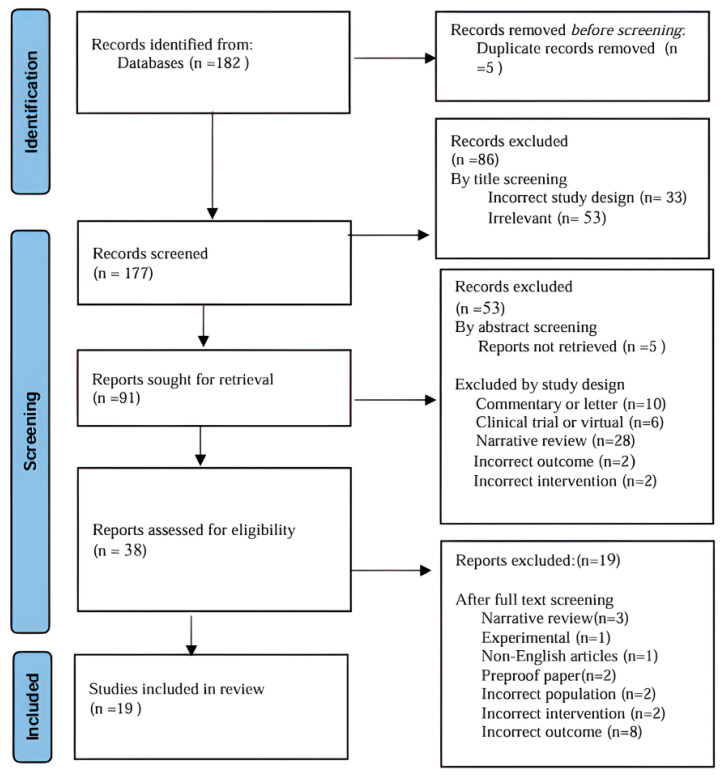
PRISMA flow diagram for selection of studies for the systematic review.

A total of 182 records were identified through systematic search. After the removal of five records as duplicates, we had 177 studies available for screening. Of those, a total of 19 observational studies were included, as shown in Figure 1, comprising nationwide registry analyses, population-based cohorts, and hospital-based retrospective or prospective studies from Europe, Asia, and North America. Sample sizes varied widely, ranging from small single-center cohorts of just over 100 patients with type 2 diabetes and COVID-19 to very large national datasets including more than 2.8 million individuals with diabetes. Most studies focused on adults with type 2 diabetes mellitus and first-episode, PCR-confirmed COVID-19, and compared patients treated with DPP-4 inhibitors (most commonly sitagliptin, linagliptin, saxagliptin, vildagliptin, or alogliptin) to non-users or users of other glucose-lowering agents (such as metformin, sulfonylureas, SGLT-2 inhibitors (sodium–glucose cotransporter-2 inhibitors), or GLP-1 receptor agonists (glucagon-like peptide-1 inhibitor). In the majority of cohorts, DPP-4 inhibitor exposure was defined as pre-existing outpatient or chronic use within a specified window prior to COVID-19 diagnosis or hospital admission, while a few studies assessed the in-hospital initiation of sitagliptin at the time of admission. Across the included studies, baseline characteristics consistently showed that DPP-4 inhibitor users were typically older and had a high prevalence of comorbidities such as hypertension, heart failure, coronary artery disease, chronic kidney disease, and chronic lung disease. The main clinical outcomes reported included COVID-19-related hospitalization or severe disease, ICU admission, and the need for non-invasive or invasive mechanical ventilation, as well as in-hospital, 28-day, or 30-day mortality and all-cause mortality. In several cohorts, additional outcomes such as hospital and ICU length of stay and time to clinical recovery were also reported. Follow-up most commonly extended to discharge, death, or 28 or 30 days after diagnosis or admission, as shown in Table 1. According to the NOS, the overall quality of included studies was mostly high with a score of 9. All the studies rated as moderate to high quality (7–9 stars) shared a similar methodological design, similar investigated outcomes and intervention. A detailed breakdown is provided in Table 2.

### 3.2. Summary of the Included Studies

Solerte and colleagues conducted a multicenter, case-control, retrospective, observational study including 338 individuals with type 2 diabetes to evaluate whether death was associated with DPP-4i use specifically in sitagliptin users among COVID-19 hospitalized patients. The results showed improved outcomes for the time to clinical endpoint (death/discharge) (HR 0.44, 95% CI 0.29–0.66, *p* = 0.0001), which is statistically significant, and a mortality rate of 18% in the sitagliptin-treated group compared to 37% in the control group, which demonstrated a lower risk of mortality among the sitagliptin-treated group [15].

Mirani and colleagues conducted a case series study including 387 individuals with COVID-19 and 90 patients with type 2 diabetes, of whom 12.2% were on DPP-4i. The study was conducted to assess the impact of antidiabetics on survival among COVID-19 patients. Among DPP-4i users, a significant reduction in mortality was observed (adjusted HR 0.13, 95% CI 0.02–0.92; *p* = 0.042) with statistical significance [16].

Pérez-Belmonte and colleagues conducted an observational multicenter nationwide cohort study with 2666 individuals who have diabetes and COVID-19, of whom 105 participants were on DPP-4i. The goal was to assess the relationship between at-home glucose-lowering medications and in-hospital mortality. Among DPP-4i users, the analysis indicated no influence on in-hospital mortality (adjusted OR 1.05; 95% CI, 0.67–2.11; *p* = 0.562), indicating a statistically non-significant result [17].

Noh and colleagues conducted a retrospective nationwide cohort study including 586 individuals with type 2 diabetes to investigate whether the usage of DPP-4i was associated with COVID-19-related outcomes, including mortality. The results demonstrated no effect of DPP-4i on all-cause mortality (adjusted HR 0.74, 95% CI 0.43–1.26), which is a statistically non-significant value [18].

Roussel and colleagues conducted a secondary analysis from a nationwide multicenter observational cohort study consisting of 2449 hospitalized diabetics, including 596 participants on DPP-4i. The aim was to evaluate the correlation between the regular use of DPP-4-i and the severity of COVID-19. The study revealed no significant effect of DPP-4i on 28-day mortality (adjusted OR 0.89, 95% CI 0.70–1.12) and a mortality rate of 18.1% compared to 21.8%, *p* = 0.0561; these values are statistically non-significant [19].

Khunti et al. conducted a nationwide English cohort study analyzing 2.85 million individuals with type 2 diabetes to evaluate whether specific glucose-lowering therapies were associated with COVID-19-related mortality. Among the total population, 479,555 individuals were prescribed DPP-4i. The findings revealed that the DPP-4i group had a higher risk of COVID-19-related mortality (HR 1.07, 95% CI 1.01–1.13) [20].

Meijer and colleagues conducted a prospective observational cohort study including 565 individuals with type 2 diabetes, of whom 28 patients were on DPP-4i. In the propensity-matched analysis, DPP-4i use showed no major effect on in-hospital death (OR 0.93, 95% CI 0.68–1.28, *p* = 0.689), which is not statistically significant [21].

Nyland and colleagues conducted a retrospective cohort study including 29,516 individuals with type 2 diabetes, of whom 2264 were treated with DPP-4i only. The study indicated no impact of DPP-4i on 28-day mortality (RR 1.03, 95% CI 0.84–1.26, *p* value = 0.78), which is not statistically significant. Importantly, the study also reported that the continuation of DPP-4i after hospitalization was associated with significantly lower mortality compared to discontinuation (matched RR 0.45, 95% CI 0.28–0.72; *p* < 0.001), demonstrating a 55% relative reduction in mortality [22].

Israelsen and colleagues conducted a population-based cohort study with a total population of 1970 individuals who had type 2 diabetes, of whom 284 individuals were on DPP-4i. The goal was to evaluate certain antidiabetics and their link to severe COVID-19 outcomes. Among DPP-4i users, the findings showed a higher 30-day mortality rate, which was more than two times higher compared with SGLT-2 inhibitor users (2.42, 95% CI 0.99–5.89) [23].

Luk and colleagues conducted a retrospective cohort population-wide study including 1220 diabetic individuals with confirmed COVID-19, of whom 199 individuals were treated with DPP-4i. The results revealed that DPP-4i had no influence on in-hospital deaths (HR 0.70, 95% CI 0.35–1.39, *p* = 0.304) [24].

Wong and colleagues conducted a retrospective territory-wide cohort study including 1214 hospitalized individuals with type 2 diabetes. The study showed no association between DPP-4i use and in-hospital deaths (fully adjusted OR 1.28, 95% CI 0.91–1.79), which is not statistically significant. In addition, the study found that the DPP-4i group had an average reduction in length of hospitalization of about 4.82 days compared to non-DPP-4i users, with a *p* value < 0.001, which is statistically significant [25].

Shestakova et al. conducted a national retrospective cohort study with a total population of 235,248 diabetic individuals with confirmed COVID-19 to assess the risk factors associated with COVID-19-related death. The results revealed that the DDP-4i group had a lower risk of death compared to the non-DDP-4i group (OR 0.59, 95% CI 0.57–0.61; *p* < 0.001), indicating a statistically significant value [1].

Ferrannini and colleagues conducted an observational longitudinal analysis of registry data including 344,413 individuals with type 2 diabetes and COVID-19, of whom 53,044 individuals were on DPP-4i, accounting for 15%. The objective was to examine the relation between glucose-lowering drugs and COVID-19 hospitalization and deaths. The findings demonstrated that the DPP-4i group had a slightly higher 30-day mortality (matched RR was 1.11, 95% CI 1.00–1.22, *p* value = 0.046) [26].

Sadid and colleagues conducted a retrospective cohort study including 220 hospitalized individuals with diabetes, of whom 44 received DPP-4i treatment, accounting for 20%. The study aims to examine the relation between DPP-4i and COVID-19 prognosis, including survival and hospital length of stay. The findings identified no significant impact of DPP-4i on survival rates (OR 0.76, 95% CI 0.13–4.41, *p* = 0.76). Moreover, DPP-4i users had a mean hospital stay of 6.57 days versus 8.03 days for non-users (*p* = 0.01), representing a statistically significant reduction in hospital stay [27].

Bramante and colleagues conducted a retrospective cohort study including 6626 individuals with diabetes. Out of the total population, DPP-4i users accounted for 12.6%. The goal is to investigate the link between glucose-lowering drugs and COVID-19 outcomes. The study focused on comparing metformin to other drug classes such as DPP-4i. The findings suggested that there was no significant difference in mortality with DPP-4i use (RR 0.82, 95% CI 0.41–1.64, *p* value = 0.581) [28].

Foresta and colleagues conducted a retrospective cohort study. The total population was 32,853 individuals with diabetes, including 4711 DPP-4i users. The objective was to evaluate whether certain antidiabetics affect COVID-19 outcomes such as mortality. Regarding DPP-4i in particular, the analysis identified a lower total mortality (RR 0.89, 95% CI 0.82–0.97), which is statistically significant [29].

Akinosoglou and colleagues conducted a prospective cohort study including 354 individuals with type 2 diabetes, of whom 134 were on DDP-4i. The objective was to evaluate outcomes in diabetic COVID-19 patients and their link to long-term glucose-lowering therapy. Among DPP-4i users, a statistically significant higher risk of in-hospital deaths was observed (fully adjusted HR 2.639, 95% CI 1.148–6.068; *p* = 0.022). Also, the mortality rate was 50.8% for the DPP-4i group compared with 35.9% among non-users with a *p*-value of 0.028 [30].

Jang and colleagues conducted a population-wide observational (retrospective) cohort study on a total population of 556 diabetics, including 358 individuals on DPP-4i. The aim was to explore the relation between specific glucose-lowering drugs and COVID-19 clinical outcomes. The findings suggested lower mortality among the DPP-4i group (fully adjusted OR 0.454, 95% CI 0.217–0.949, *p*-value = 0.036), indicating a statistically significant result [31].

Park and colleagues conducted a retrospective nationwide cohort study including 16,134 individuals hospitalized with diabetes to evaluate whether DPP-4i medications were associated with mortality among COVID-19 patients. The analysis demonstrated a decreased 30-day mortality rate among DPP-4i users (HR 0.455, 95% CI 0.414–0.499), which is statistically significant [32].

### 3.3. Clinical Outcomes

The primary outcome was mortality, and the secondary outcomes included length of hospital stay, ICU admission, and mechanical ventilation. With respect to mortality, it was reported differently in terms of effect measures such as hazard ratio, risk ratio and odd ratios. Also, there were different time points in which mortality was evaluated, including in-hospital, 30-day, and all-cause mortality. Collectively, a total of eight studies found no association with mortality, while seven studies (including Nyland et al., 2021 [22]) observed a decrease and four studies identified a higher risk. Although Nyland et al. found a neutral effect on 28-day mortality, a significant benefit was observed with continued in-hospital use.

**Figure 2:**
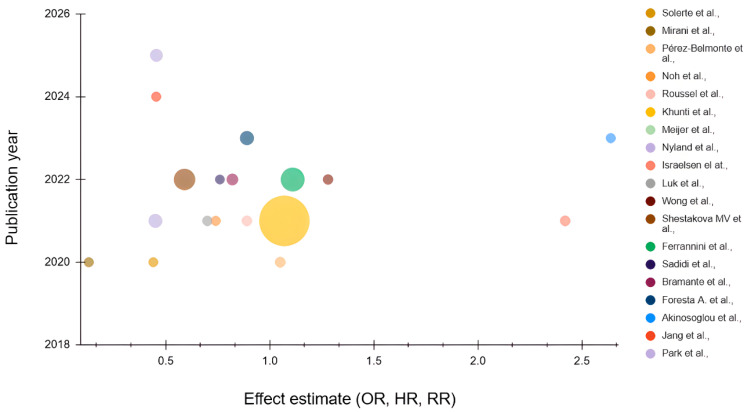
Distribution of reported mortality effect estimates across included studies. Each bubble represents an individual study. The x-axis displays reported mortality effect estimates (OR, HR, or RR) as published, and the y-axis indicates year of publication. Bubble size is proportional to study sample size. Colors distinguish individual studies. Estimates are presented descriptively and were not pooled. (Solerte et al., 2020 [15]; Mirani et al., 2020 [16]; Pérez-Belmonte et al., 2020 [17]; Noh et al., 2021 [18]; Roussel et al., 2021 [19]; Khunti et al., 2021 [20]; Meijer et al., 2021 [21]; Nyland et al., 2021 [22]; Israelsen et al., 2021 [23]; Luk et al., 2021 [24]; Wong et al., 2022 [25]; Shestakova et al., 2022 [1]; Ferrannini et al., 2022 [26]; Sadidi et al., 2022 [27]; Bramante et al., 2022 [28]; Foresta et al., 2023 [29]; Akinosoglou et al., 2023 [30]; Jang et al., 2024 [31]; Park et al., 2025 [32]).

Regarding the secondary outcomes, only two studies reported length of hospital stay, seven studies reported ICU admission, and eight reported mechanical ventilation. All the outcomes are reported in detail for each individual study in Table 3 and Figure 2, which demonstrate the distribution of reported mortality effect estimates across included studies.

To conclude, most of the findings suggest no negative impact of DPP-4i on the primary and secondary outcomes; on the other hand, a considerable proportion of studies showed beneficial outcomes.

## 4. Discussion

This systematic review assessed the association between DPP-4 inhibitor use and clinical outcomes among patients with diabetes who developed COVID-19. Even though the included studies varied in methodology, size, and geographical setting, several overarching findings emerged. The evidence suggests that DPP-4 inhibitors do not confer an increased risk of adverse COVID-19 outcomes [17,18,19,21,24,25,27,28]. In several cohorts, their use was associated with improved survival or reduced clinical deterioration [1,15,16,22,29,31,32].

The mortality effect of DPP-4 inhibitors among diabetic patients with COVID-19 emerges as largely neutral to favorable instead of being consistently negative when the data are integrated. Even though there was a subset of studies that found DPP-4 inhibitors were associated with an increased risk of mortality, this was not linked directly to the use of the DPP-4 inhibitors [20,23,26,30]. The authors suggested that the results were due to patients tending to be older, having more advanced diabetes, or being immunocompromised. On the other hand, several large cohort studies assessed the continued use of DPP-4 inhibitors and showed a significant reduction in mortality and clinical outcomes [1,22,29,32]. Nevertheless, most of these studies did not show any significant difference in mortality outcomes, particularly those utilizing statistical adjustment. 

A major strength of this systematic review is the comprehensive inclusion of data from diverse geographical regions and multiple healthcare systems, which enhances the generalizability of the findings. The review incorporates evidence from large population-based cohorts, national registries, and multicenter hospital studies, allowing a broad assessment of DPP-4 inhibitor use across different clinical contexts. Another strength is the detailed extraction of outcomes beyond mortality, such as ICU admission, mechanical ventilation, and length of stay—which provides a more nuanced understanding of disease severity. Furthermore, several of the included studies applied robust statistical techniques, including propensity score matching, inverse probability weighting, and multivariable modeling, helping to mitigate confounding. However, several limitations should be acknowledged.

First, variations in exposure timing across studies represent a main source of heterogeneity. One of the cohorts defined DPP-4 inhibitor use as 90 days preceding COVID-19 diagnosis [23], while other studies did not specify an exact exposure window, instead referring broadly to pre-admission or pre-infection use. Additionally, in certain cohorts, DPP-4 inhibitors were initiated in the hospital rather than prior to admission, which may reflect acute clinical deterioration and thus be associated with more severe disease and unfavorable outcomes. In contrast, pre-admission initiation may be associated with more favorable outcomes, potentially reflecting a longer duration of therapy or differences in baseline health status.

Secondly, the comparator groups might be widely different due to residual confounding, which may affect bias in either direction. Although adjustments were made for certain comorbidities, not all studies reported key baseline variables or accounted for other important confounders such as diabetes severity, duration, glycemic control at admission, or concomitant treatment. If patients with poor health status or more advanced diabetes were distributed unequally among comparator groups, this may reflect disease severity instead of treatment effect.

Another major and related factor seen in some studies is the use of active comparators, for example, SGLT-2 inhibitors or metformin. Depending on the agent of the active comparator, it may exert specific independent pharmacological effects in the comparator group, which may exaggerate both harmful and neutral effects of DPP-4i.

Third, outcome definitions were not always standardized, particularly for mortality. Mortality definitions in most studies were inconsistent with apparent variations. Part of the included studies referred to mortality as all-cause mortality, while some cohorts provided in-hospital death, 30-day mortality, or 28-day mortality. The diversity in mortality definitions creates a barrier to the unity of the interpreted results and whether the measured outcome is the same across all studies. Heterogeneity in follow-up duration, testing criteria, and hospitalization thresholds further complicates interpretation.

Fourth, heterogeneity related to the pandemic phases should be considered. A considerable number of studies were conducted during different phases of the pandemic. Early in the pandemic, hospitalization criteria were less clearly defined, and clinical management was less standardized compared with later waves. Changes in clinical practice, treatment protocols, resource availability, and thresholds for COVID-19 testing introduced methodological differences representing key sources of heterogeneity and may have contributed to inconsistencies. Also, the selection of the given treatment was based on standard clinical practice in contrast to random selection seen in random clinical trials; all of this introduces a potential of confounding by indication. Moreover, the independent effect of DPP-4 inhibitor use could not be adequately assessed because of the lack of uniform comparator groups. As indicated in Table 2, most studies were rated as high quality according to the Newcastle–Ottawa Scale (NOS); however, this rating does not eliminate the risk of bias. The NOS does not evaluate causality, cannot fully address confounding variables, and may not guarantee that the study population is representative. Furthermore, exposure ascertainment may be susceptible to bias, as the NOS does not assess medication adherence or correct dosing. Therefore, even studies rated as high quality may remain subject to bias.

Finally, publication bias cannot be excluded when interpreting the results. The studies demonstrating strong associations, either beneficial or harmful, may be more likely to be published. Accordingly, although the current review provides reassurance regarding the safety of DPP-4 inhibitors in patients with diabetes and COVID-19, the findings should be interpreted with caution, and variations in outcome definitions should be taken into consideration. All included studies were observational; therefore, causality cannot be inferred and randomized clinical trials are required before any causal or therapeutic benefit can be inferred.

## 5. Conclusions

In conclusion, the findings of this review reinforce the safety of DPP-4 inhibitors in the context of COVID-19 and highlight the importance of individualized therapy based on each patient’s clinical status. However, the current evidence, derived from heterogeneous observational studies, is insufficient to conclude any beneficial effects of DPP-4 inhibitors. Randomized controlled trials (RCTs) would be the most reliable approach to determine any potential protective effects of these medications and to confirm their safety.

## Figures and Tables

**Table 1:** Baseline characteristic.

Author/Year	Setting (Country/Registry)	Study Design	Total Number of Participants	Mean/Median Age (Years)	DPP4i Use Defined As	Comparator
Solerte et al., 2020 [15]	Northern Italy	Multicenter, Case-Control, Retrospective, Observational	338	Sitagliptin group 69 ± 1.0	Sitagliptin given at hospital admission	Standard of care (including insulin)
Mirani et al., 2020 [16]	Humanitas Clinical and Research Hospital, IRCCS, Milan, Lombardy, Italy	Case series	387	66 years (IQR 54–76)	Outpatient use continued during hospitalization	Non-DPP-4i users
Pérez-Belmonte et al., 2020 [17]	Spain	Observational multicenter nationwide cohort study	2666	Mean ± SD 74.9 ± 8.4	Outpatient chronic use before hospital admission (documented in medical record)	Other glucose-lowering drugs (metformin, insulin, SGLT-2i, GLP-1RA, etc.)
Noh et al., 2021 [18]	South Korea	Retrospective nationwide cohort	586	NR	Outpatient chronic prescription	Second/third-line antidiabetic drugs
Roussel et al., 2021 [19]	France	Secondary analysis from nationwide multicenter observational cohort	2449	Mean ± SD 70.9 ± 12.5	Chronic routine before hospitalization	Non-DPP-4i users
Khunti et al., 2021 [20]	England	Nationwide retrospective cohort	2.85 million	Median 67 years (IQR 57–77)	Prescribed by GP before pandemic	Non-DPP-4i users
Meijer et al., 2021 [21]	Netherlands	Prospective observational cohort	565	DPP-4i users Mean ± SD 66.88 ± 12.41	Outpatient chronic therapy before hospitalization	Non-DPP-4i users
Nyland et al., 2021 [22]	Multinational	Retrospective cohort study	29,516	Mean (SD) 60.9 (15.0)	Use of DPP-4 inhibitors only in the prior 6 months before first COVID-19 record	Non-DPP-4i users
Israelsen et al., 2021 [23]	Denmark	Population-based cohort study	1970	DPP-4i users: median 67 (IQR 57–76)	Redeemed prescription of DPP-4 inhibitor within 90 days before SARS-CoV-2 positive test	SGLT-2i (active comparator)
Luk et al., 2021 [24]	Hong Kong	Retrospective cohort (population-wide)	1220	Median age 65.3	Prescription record of the DPP-4 inhibitor drug within 12 months before admission; no minimum exposure time set	Non-DPP-4i users
Wong et al., 2022 [25]	Hong Kong	Retrospective territory-wide cohort	1214	DPP4i users Mean ± SD 66.3 ± 11.7	Chronic outpatient therapy continued in-hospital	Non-DPP-4i users
Shestakova et al., 2022 [1]	Russia	Nationwide retrospective cohort	235,248	NR	Initiated at admission (not home use)	Non-DPP-4i users
Ferrannini et al., 2022 [26]	Sweden	Observational study	344,413	Median IQR, 72 (62–79)	use of DPP4 inhibitors as having a prescription for any of the listed ATC codes between 1 September 2019, and 1 February 2020	Non-DPP-4i users
Sadidi et al., 2022 [27]	Iran	Retrospective cohort	220	Mean ± SD 66.13 ± 12.3 yrs	Chronic use ≥ 1 year before infection	SGLT-2 inhibitors (active comparator)
Bramante et al., 2022 [28]	US	Retrospective cohort	6626	Mean ± SD 60.7 ± 12.0	Pre-existing use of DPP4I within the 90 days prior to the +SARS-CoV-2 result	Metformin monotherapy (active comparator)
Foresta et al., 2023 [29]	Italy	Retrospective cohort study	32,853	Mean (SD) 71.86 (11.97)	At least 2 prescriptions of DPP-4i prior to or at the time of COVID-19 diagnosis	Non-DPP-4i users
Akinosoglou et al., 2023 [30]	Greece	Prospective cohort study	354	Median age 70 years (IQR 62–79)	Prior chronic use of DPP4 inhibitors as part of T2D management (at hospital admission)	Non-DPP-4i users
Jang et al., 2024 [31]	Korea	Population-wide observational (retrospective) cohort	556	NR	DPP-4i use, within the year before the COVID-19 diagnosis	Non-DPP-4i users
Park et al., 2025 [32]	South Korea	Retrospective nationwide cohort	16,134	Mean ± SD 69.19 ± 13.99	Outpatient chronic prescription before hospitalization	Non-DPP-4i users

NR = not reported. DPP-4i = dipeptidyl peptidase-4 inhibitors, SGLT2 = sodium–glucose cotransporter-2, GLP-1RA = glucagon-like peptide-1 receptor agonist, GP = general practitioner, COVID-19 = coronavirus disease 2019, IQR = interquartile range, SD = standard deviation.

**Table 2:** Quality assessment of included studies using the Newcastle–Ottawa Scale (NOS).

Author/Year	Selection (0–4)	Comparability (0–2)	Outcome (0–3)	Total (0–9)	Quality
Solerte et al., 2020 [15]	★★★	★★	★★★	8	High
Mirani et al., 2020 [16]	★★★	★★	★★★	8	High
Pérez-Belmonte et al., 2020 [17]	★★★★	★★	★★★	9	High
Noh et al., 2021 [18]	★★★★	★★	★★★	9	High
Roussel et al., 2021 [19]	★★★★	★★	★★★	9	High
Khunti et al., 2021 [20]	★★★★	★★	★★★	9	High
Meijer et al., 2021 [21]	★★★★	★★	★★★	9	High
Nyland et al., 2021 [22]	★★★★	★★	★★★	9	High
Israelsen et al., 2021 [23]	★★★★	★★	★★★	9	High
Luk et al., 2021 [24]	★★★★	★	★★★	8	High
Wong et al., 2022 [25]	★★★★	★★	★★★	9	High
Shestakova et al., 2022 [1]	★★★★	★	★★★	8	High
Ferrannini et al., 2022 [26]	★★★★	★★	★★★	9	High
Sadidi et al., 2022 [27]	★★★	★	★★★	7	Moderate
Bramante et al., 2022 [28]	★★★★	★★	★★★	9	High
Foresta et al., 2023 [29]	★★★★	★★	★★★	9	High
Akinosoglou et al., 2023 [30]	★★★★	★★	★★★	9	High
Jang et al., 2024 [31]	★★★★	★★	★★★	9	High
Park et al., 2025 [32]	★★★★	★★	★★★	9	High

The NOS evaluates studies on Selection (0–4 stars), Comparability (0–2 stars), and Outcome (0–3 stars). Stars (★) indicate the score per domain, with the total score reflecting overall study quality.

**Table 3:** Summary of clinical outcome effect estimates across included studies.

Outcome	Study (Author, Year)	Effect Estimate (95% CI) aHR, OR,	*p*-Value	Effect
Mortality Definitions				
A time to clinical endpoint (death/discharge) 30-day mortality	Solerte et al., 2020 [15]	HR 0.44 (95% CI 0.29–0.66) 18% deaths in sitagliptin-treated group vs. 37% (control)	0.0001	Decrease
Mortality risk	Mirani et al., 2020 [16]	HR 0.13 (95% CI 0.02–0.92)	0.042	Decrease
In-hospital mortality	Pérez-Belmonte et al., 2020 [17]	OR 1.05 (95% CI 0.67–2.11)	0.562	No effect
All-cause mortality	Noh et al., 2021 [18]	HR 0.74 (95% CI 0.43–1.26)		No effect
28-day mortality	Roussel et al., 2021 [19]	OR 0.89 (95% CI 0.70–1.12) (18.1% vs. 21.8%; *p* = 0.0561)	0.0561	No effect
COVID-19-related mortality	Khunti et al., 2021 [20]	HR 1.07 (95% CI 1.01–1.13)		Increase
In-hospital deaths	Meijer et al., 2021 [21]	OR 0.93 (95% CI 0.68–1.28)	0.689	No effect
28-days mortality Continued use after hospitalization	Nyland et al., 2021 [22]	RR 1.03 (95% CI 0.84–1.26) RR 0.45 (0.28–0.72)	0.78 <0.001	No effect Decrease
30-day mortality	Israelsen et al., 2021 [23]	RR 2.42 (95% CI 0.99–5.89)		Increase
In-hospital death	Luk et al., 2021 [24]	HR 0.70 (95% CI 0.35 to 1.39)	0.304	No effect
In-hospital death	Wong et al., 2022 [25]	OR 1.28 (95% CI 0.91–1.79)	0.151	No effect
COVID-19-related fatality	Shestakova et al., 2022 [1]	OR 0.59 (95% CI: 0.57–0.61)	<0.001	Decrease
30-day mortality	Ferrannini et al., 2022 [26]	RR 1.11 (95% CI 1.00–1.22)	0.046	Increase
Survival rates	Sadidi et al., 2022 [27]	OR 0.76 (95% CI 0.13–4.41)	0.76	No effect
In-hospital and before-hospital mortality	Bramante et al., 2022 [28]	RR 0.82 (95% CI 0.41–1.64)	0.581	No effect
Total mortality	Foresta A. et al., 2023 [29]	RR 0.89 (95% CI 0.82–0.97)		Decrease
28-day mortality	Akinosoglou et al., 2023 [30]	HR 2.639,95% (95% CI 1.148–6.068)	0.022	Increase
Death	Jang et al., 2024 [31]	OR 0.454 (95% CI 0.217–0.949)	0.036	Decrease
30-day all-cause mortality	Park et al., 2025 [32]	HR 0.455 (95% CI 0.414–0.499)		Decrease
Hospital length of stay				
	Wong et al., 2022 [25]	(−4.82 days, 95% CI −6.80 to −2.84)	<0.001	Decrease
	Sadidi et al., 2022 [27]	6.57 ± 2.3 vs. 8.03 ± 4.4 days;	0.01	Decrease
ICU admission				
	Solerte et al., 2020 [15]	HR:0.51 (95% CI 0.27–0.95)	0.03	Decrease
	Meijer et al., 2021 [21]	OR 0.93 (95% CI 0.68–1.28)	0.689	No effect
	Israelsen el at., 2021 [23]	RR 1.30 (95% CI 0.54–3.12)		No effect
	Luk et al., 2021 [24]	HR 0.45 (95% CI 0.28 to 0.74)	0.002	Decrease
	Akinosoglou et al., 2023 [30]	OR 2.524 (95% CI 1.217–5.232),	0.013	Increase
	Jang et al., 2024 [31]	OR 0.959 (95% CI 0.564–1.631)	0.877	No effect
	Park et al., 2025 [32]	14.0% vs. 16.6%		Decrease
Mechanical ventilation				
	Solerte et al., 2020 [15]	HR 0.27 (95% CI 0.11–0.62)	0.003	Decrease
	Roussel et al., 2021 [19]	OR 0.97 (95% CI 0.77–1.23)		No effect
	Meijer et al., 2021 [21]	OR 0.98 (95% CI 0.81–1.19)	0.911	No effect
	Luk et al., 2021 [24]	HR 0.57 (95% CI 0.29 to 1.11)	0.098	No effect
	Israelsen el at., 2021 [23]	RR 2.22 (95% CI 0.77–6.46)		No effect
	Wong et al., 2022 [25]	OR 0.30 (95% CI 0.21–0.42)	<0.001	Decrease
	Bramante et al., 2022 [28]	RR 0.68 (95% CI 0.32–1.44)	0.315	No effect
	Jang et al., 2024 [31]	OR 1.090 (95% CI 0.430–2.762)	0.856	No effect

## Data Availability

All data generated or analyzed for this paper are included in this article.

## Supplementary Materials

The following supporting information can be downloaded at https://www.mdpi.com/article/10.3390/jcm15062117/s1: PRISMA 2020 checklist.

## Abbreviations

The following abbreviations are used in this manuscript:

DPP-4i	Dipeptidyl Peptidase-4 Inhibitor
COVID-19	Coronavirus Disease 2019
SARS-CoV-2	Severe Acute Respiratory Syndrome Coronavirus 2
IL	Interleukin
ICU	Intensive Care Unit
NOS	Newcastle–Ottawa Scale
PRISMA	Preferred Reporting Items for Systematic Reviews and Meta-Analyses
PCR	Polymerase Chain Reaction
SGLT-2i	Sodium–Glucose Cotransporter-2 inhibitors
GLP-1	Glucagon-Like Peptide-1
GP	General Practitioner
HR	Hazard Ratio
OR	Odds Ratio
RR	Relative Risk
CI	Confidence Interval
1QR	Interquartile Range
SD	Standard Deviation
